# Supplementary material for: Achieving stable dynamics in neural circuits
Source: PLoS Comput Biol. 2020 Aug 7;16(8):e1007659. doi: 10.1371/journal.pcbi.1007659 (PMC7446801; doi:10.1371/journal.pcbi.1007659)
Supplement: S1 Text — We kept the appendix self-contained by restating the basic results of contraction analysis and linear algebra which we used often in our proofs. (PDF) [file pcbi.1007659.s001.pdf]

Appendix to ‘Achieving Stability in Neural Circuits’

Leo Kozachkov[1,2,3], Mikael Lundqvist *[1,2], Jean-Jacques Slotine[1,3], and Earl K. Miller[1,2]

[1]Department of Brain & Cognitive Sciences, Massachusetts Institute of Technology (MIT), 43 Vassar Street, Cambridge, MA 02139, USA

[2]The Picower Institute for Learning & Memory, MIT, 43 Vassar Street, Cambridge, MA 02139, USA

[3]Nonlinear Systems Laboratory, MIT, Cambridge, Massachusetts 02139, USA.

Contents

1 Preliminaries **3**

1.1 Mathematical Preliminaries 3

1.1.1 Matrix Notions 3

1.1.2 Matrix Measures 4

1.1.3 Matrix Vecorization 5

1.2 Basic Contraction Definition 6

*Also first author

2 Contracting Dynamics of A Leaky Neuron **7**
2.1 A Motivating Example 7
2.2 Leaky Neuron with No Autapse 8

3 Synaptic Dynamics that Favor or Preserve Contraction **9**
3.1 Anti-Hebbian Dynamics Produce Contraction 11
3.1.1 Off-Diagonal Blocks of the Jacobian 12
3.1.2 Diagonal Blocks of the Jacobian 14

4 Sparsity Ensures Contraction **16**

5 Contraction in RNNs with Static Weights **18**
5.0.1 Contraction in Circuits with Perfect E-I Balance 19
5.0.2 Contraction in Circuits With Statistical E-I Balance . . . 20
5.1 Relationship to Echo State Networks 23

1 Preliminaries

1.1 Mathematical Preliminaries

1.1.1 Matrix Notions

We will denote vectors and matrices with bold font. For example: $\mathbf{x} \in \mathbb{R}^N$ is an N-dimensional column vector and $\mathbf{A} \in \mathbb{R}^{N \times M}$ is an $N \times M$ matrix. We use the notation $\mathbf{P} \succ 0$ to denote a positive definite matrix, which is a square matrix such that, for all $\mathbf{x} \in \mathbb{R}^N \neq \mathbf{0}$, the quadratic form $\mathbf{x}^T\mathbf{P}\mathbf{x}$ is strictly positive:

$$\mathbf{P} \succ 0 \equiv \mathbf{x}^T\mathbf{P}\mathbf{x} > 0 \tag{1.1.1}$$

When the quadratic form is non-negative (i.e $\mathbf{x}^T\mathbf{P}\mathbf{x} \geq 0$) then we say that that $\mathbf{P}$ is positive semi-definite and write $\mathbf{P} \succcurlyeq 0$. Negative definiteness and semi-definiteness are defined the same way, with the sign of the above inequalities reversed.

Every real, square matrix $\mathbf{Q}$ can be decomposed into a symmetric part and a skew symmetric part. We denote the symmetric part of $\mathbf{Q}$ as $\mathbf{Q}_s$ and define it as follows

$$\mathbf{Q}_s \equiv \frac{\mathbf{Q} + \mathbf{Q}^T}{2} \tag{1.1.2}$$

and the antisymmetric (skewsymmeric) part as

$$\mathbf{Q}_a \equiv \frac{\mathbf{Q} - \mathbf{Q}^T}{2} \tag{1.1.3}$$

Note that $\mathbf{Q} = \mathbf{Q}_s + \mathbf{Q}_a$. Also note that without ambiguity, when we say that a non-symmetric $\mathbf{Q}$ is positive definite, we mean that $\mathbf{Q}_s \succ 0$. This is because:

$$\mathbf{x}^T\mathbf{P}\mathbf{x} = \mathbf{x}^T\mathbf{P}_s\mathbf{x}$$

Substituting this equality back into (1.1.1), we see that a matrix is positive definite if and only if its symmetric part is positive definite. For more informa-

tion on positive definite matrices, see section 7.1 of [3] or section 3.5.1 of [5], for example.

1.1.2 Matrix Measures

We will also need the fact that a given vector norm $|\cdot|$ will induce a matrix norm $||\cdot||$

$$||\mathbf{Q}|| \equiv \sup_{|x|=1} |\mathbf{Qx}|$$

as well as a matrix measure $\mu(\cdot)$

$$\mu(\mathbf{Q}) \equiv \lim_{h\to 0} \frac{||\mathbf{I} - h\mathbf{Q}|| - 1}{h}$$

where $\mathbf{I}$ denotes the identity matrix. Different norms induce different matrix measures, but all matrix measures share certain common properties. In particular, for any matrix measure μ we have subadditivity:

$$\mu(\mathbf{X} + \mathbf{Y}) \leq \mu(\mathbf{X}) + \mu(\mathbf{Y}) \tag{1.1.4}$$

positive homogeneity:

$$\forall c \geq 0, \quad \mu(c\mathbf{X}) = c\mu(\mathbf{X}) \tag{1.1.5}$$

as well as

$$\mu(\mathbf{I}) = 1 \;\text{ and }\; \mu(-\mathbf{I}) = -1 \tag{1.1.6}$$

For more information about matrix measures the reader is referred to section 2.2.2 of [7] or section 2 of [1].In this paper we will rely primarily on the matrix measures induced by the infinity-norm $|\cdot|_\infty$ and the 2-norm $|\cdot|_2$. These norms induce the matrix measures

$$\mu_\infty(\mathbf{Q}) = \sup_i (Q_{ii} + \sum_{j\neq i} |Q_{ij}|) \tag{1.1.7}$$

$$\mu_2(\mathbf{Q}) = \lambda_{\max}(\mathbf{Q}_s) \tag{1.1.8}$$

Finally, consider the (potentially time-varying), non-singular matrix $\mathbf{\Theta}$ and the associated weighted 2-norm and weighted infinity-norm : $|\mathbf{x}|_{\Theta,2} = |\mathbf{\Theta x}|_2$. The matrix measures induced by these norms are:

$$\mu_{2,\Theta}(\mathbf{Q}) = \mu_2(\dot{\mathbf{\Theta}}\mathbf{\Theta}^{-1} + \mathbf{\Theta Q \Theta}^{-1}) \tag{1.1.9}$$

$$\mu_{\infty,\Theta}(\mathbf{Q}) = \mu_\infty(\dot{\mathbf{\Theta}}\mathbf{\Theta}^{-1} + \mathbf{\Theta Q \Theta}^{-1}) \tag{1.1.10}$$

1.1.3 Matrix Vecorization

Finally, consider an arbitrary $N \times N$ matrix $\mathbf{A}$. The vectorization of $\mathbf{A}$, which we denote by $\mathbf{v}_A$, is the $N^2 \times 1$ column vector obtained by stacking the columns of $\mathbf{A}$ on top of one another, starting from the leftmost column and ending at the rightmost column. Consider the example of:

$$\mathbf{A} = \begin{bmatrix} a & b \\ c & d \end{bmatrix}$$

then

$$\mathbf{v}_A = \begin{bmatrix} a \\ c \\ b \\ d \end{bmatrix}$$

We also define the $N^2 \times N^2$ matrix $\mathbf{D}_A$ as the diagonal matrix with the elements of $\mathbf{A}$ along its diagonal, starting with the leftmost column of $\mathbf{A}$ and ending with the rightmost column. For the $\mathbf{A}$ matrix defined above, we have:

$$\mathbf{D}_A = \begin{bmatrix} a & 0 & 0 & 0 \\ 0 & c & 0 & 0 \\ 0 & 0 & b & 0 \\ 0 & 0 & 0 & d \end{bmatrix}$$

These two operators have a special relationship to the Hadamard product (element-wise matrix multiplication). Consider a matrix $\mathbf{Z}$ defined as:

$$\mathbf{Z} = \mathbf{X} \odot \mathbf{Y}$$

where $\mathbf{X}$ and $\mathbf{Y}$ are of the same size. Then we have the following relationship:

$$\mathbf{v}_Z = \mathbf{D}_X \mathbf{v}_Y \tag{1.1.11}$$

which can be verified through direct inspection.

1.2 Basic Contraction Definition

In this section we define what a contracting system is, and state several properties of these systems that will be useful for the remainder of the supplementary. The reader is referred to [4] and [6] for more details. Consider a deterministic system of the form

$$\dot{\mathbf{x}} = \mathbf{f}(\mathbf{x}, t) \tag{1.2.1}$$

where $\mathbf{f}$ is an $N{\times}1$ nonlinear vector function and $\mathbf{x}$ is the $N{\times}1$ state vector. We assume that all quantities are real and smooth, by which we mean that any required derivative or partial derivatives exist and are continuous. Denote the $N \times N$ time-varying Jacobian of this system as $\mathbf{J}(\mathbf{x}, t) = \mathbf{J} = \frac{\partial \mathbf{f}}{\partial \mathbf{x}}$.

If there exists a matrix measure μ such that

$$\forall \mathbf{x}, t \quad \mu(\mathbf{J}) \leq -\lambda \;\text{ with }\; \lambda > 0 \tag{1.2.2}$$

then all trajectories of (1.2.1) converge exponentially towards one another with rate λ and the system is said to be *contracting*. In particular, let $R(t) =$

$|\mathbf{x}(t) - \mathbf{x}'(t)|$ denote the distance between any two trajectories of system (1.2.1) and assume the system is contracting. Then there exists a constant $k \geq R(0)$ such that

$$0 \leq R(t) \leq ke^{-\lambda t}$$

To avoid ambiguity in language, we now define some terms associated with the measures μ_1,μ_2 and μ_∞ . As in [4], consider a metric $\mathbf{M} = \mathbf{\Theta}^T\mathbf{\Theta} \succ 0$ and the following associated quantity, called the *generalized Jacobian*:

$$\mathbf{F} = \dot{\mathbf{\Theta}}\mathbf{\Theta}^{-1} + \mathbf{\Theta}\mathbf{J}\mathbf{\Theta}^{-1} \tag{1.2.3}$$

If the matrix measures induced by the 1,2, or infinity norm are negative definite when applied to $\mathbf{F}$, i.e.

$$\mu_i(\mathbf{F}) < -\lambda \text{ with } i = 1, 2, \text{ or } \infty \tag{1.2.4}$$

then we say that the system is contracting in metric $\mathbf{\Theta}$ with rate λ. Calling $\mathbf{\Theta}$ a metric is a slight abuse of language, because the real metric is $\mathbf{M} = \mathbf{\Theta}^T\mathbf{\Theta}$, but since we will always use the symbol $\mathbf{\Theta}$ in the generalized Jacobian, this won't cause any confusion.

2 Contracting Dynamics of A Leaky Neuron

In this section we apply contraction analysis to the dynamics of a single, leaky, input-driven neuron. Analyzing the case of one neuron will turn out to be helpful when thinking about the case of many neurons.

2.1 A Motivating Example

Consider the activation of a pure integrator neuron that is receiving constant input, I:

$$\dot{x} = I \tag{2.1.1}$$

This differential equation can be solved analytically, and has the solution:

$$x(t) = It + x(0) \tag{2.1.2}$$

Since the solution does not forget x(0), its initial condition, this system is not contracting. However, if we were to add a 'leak' term, for example $-x$ to (2.1.1), we would have

$$\dot{x} = -x + I \tag{2.1.3}$$

which yields the solution

$$x(t) = I + \chi e^{-t} \tag{2.1.4}$$

with $\chi = x(0) - I$. Since this system forgets $x(0)$ exponentially quickly, we may conclude that it is contracting (of course we also could have computed the Jacobian, which in the case of (2.1.3) is -1). In the case of nonlinear dynamics and time-varying input, we will not be able to solve for the dynamics of x explicitly to determine contracting properties–instead, we will have to analyze the Jacobian.

2.2 Leaky Neuron with No Autapse

Let x denote the activation of a single, isolated, leaky-integrator neuron. Assume that this neuron has no self-connection (i.e no autapse) and obeys the equation:

$$\tau\dot{x} = -\alpha x + I(t) \tag{2.2.1}$$

where $\tau > 0$ is the time-constant of integration, $\alpha > 0$ is the time-constant of the 'leak', and $I(t)$ is an external input into the neuron. If $\alpha = 0$ this would be a pure integrator neuron (see section 2.1).Dividing through by τ, we may rewrite (2.2.1) as

$$\dot{x} = -\frac{\alpha}{\tau}x + \frac{1}{\tau}I(t)$$

the Jacobian of this scalar system is:

$$J = \frac{\partial \dot{x}}{\partial x} = -\frac{\alpha}{\tau} \tag{2.2.2}$$

since α and τ are positive, $\frac{\alpha}{\tau}$ is also positive, which means that $-\frac{\alpha}{\tau}$ is negative. Since the Jacobian is negative, we can conclude that the isolated leaky neuron is contracting for all inputs. In the paper we will consider several classes of leaky neurons, including neurons that obey the equation

$$\tau\dot{x} = h(x) + I(t) \tag{2.2.3}$$

where $h(x)$ is a nonlinear function that satisfies

$$\exists\, \beta > 0, \quad \forall\, x, \quad \forall\, t \geq 0, \quad \frac{\partial h}{\partial x} \leq -\beta < 0 \tag{2.2.4}$$

By the same arguments as above, leaky neurons of this form are also contracting in isolation. Of course, the standard leak term of $-x$ is a special case of $h(x)$. For $h(x) = -x$ we find that $\beta = 1$.

3 Synaptic Dynamics that Favor or Preserve Contraction

The assumption that synaptic dynamics are local introduces strong structure into the Jacobian of the overall network. In particular, it implies that the 'synaptic' diagonal block of the Jacobian will be a diagonal matrix. We'll now establish some simple facts about matrices with this structure that will be useful later on.

Theorem 1. *Consider the following matrix*

$$\mathbf{F} = \begin{bmatrix} \mathbf{X} & \mathbf{GA} \\ -\mathbf{BG}^T & \mathbf{D} \end{bmatrix}$$

where $\mathbf{X}$ *is an arbitrary negative definite matrix,* $\mathbf{D}$ *is diagonal and negative definite,* $\mathbf{A}$ *and* $\mathbf{B}$ *are diagonal and positive definite and* $\mathbf{G}$ *is arbitrary. Then there exists a diagonal metric* $\mathbf{\Theta}$ *such that*

$$\mathbf{\Theta F \Theta}^{-1} \prec 0 \tag{3.0.1}$$

Proof. Consider the candidate metric

$$\mathbf{\Theta} = \begin{bmatrix} \mathbf{I} & \mathbf{0} \\ \mathbf{0} & \mathbf{L} \end{bmatrix}$$

where $\mathbf{L}$ is some nonsingular matrix, to be determined. Applying this to the matrix $\mathbf{F}$, we find that

$$\mathbf{\Theta F \Theta}^{-1} = \begin{bmatrix} \mathbf{X} & \mathbf{GAL}^{-1} \\ -\mathbf{LBG}^T & \mathbf{D} \end{bmatrix}$$

We would like the off-diagonal terms of this matrix to cancel out when taking the symmetric part. To make this happen, we need

$$\mathbf{BL} = \mathbf{AL}^{-1}$$

Which can be easily manipulated into

$$\mathbf{L}^2 = \mathbf{AB}^{-1}$$

and thus

$$\mathbf{L} = \sqrt{\mathbf{AB}^{-1}}$$

The square root is guaranteed to exist because $\mathbf{A}$ and $\mathbf{B}$ are both positive definite and diagonal by assumption. Taking the symmetric part of (3.0.1), we find that:

$$\mathbf{\Theta F \Theta}^{-1} + (\mathbf{\Theta F \Theta}^{-1})^T = \begin{bmatrix} \mathbf{X} + \mathbf{X}^T & 0 \\ 0 & 2\mathbf{D} \end{bmatrix}$$

which is negative definite since the diagonal blocks are negative definite by assumption. This proves the result.

□

We will use this result in the next section to show that anti-hebbian plasticity gives rise to contracting dynamics.

3.1 Anti-Hebbian Dynamics Produce Contraction

Consider RNNs of the following form:

$$\dot{\mathbf{x}} = \mathbf{f}(\mathbf{x}, \mathbf{W}, t) = \mathbf{h}(\mathbf{x}) + \mathbf{W}\mathbf{x} + \mathbf{u}(t)$$

Where $h_i(x_i)$ is a leak-term (see (2.2.4) for definition), $\mathbf{W}$ is the weight matrix of the network, and $\mathbf{u}(t)$ is a time-varying input. And consider the plasticity described in the main text,given by

$$\dot{W}_{ij} = -k_{ij} x_i x_j - \gamma(t) W_{ij}$$

where the matrix of k_{ij} terms, $\mathbf{K}$ must be positive semi-definite for reasons to be discussed. To prove that this plasticity leads to overall contracting dynamics, we need to show that the overall Jacobian of the system is contracting in some metric. We will do this in several steps. To give an overview of the strategy, first write the synaptic dynamics as an $N^2 \times 1$ vector:

$$\dot{\mathbf{v}}_W = \mathbf{g}(\mathbf{v}_W, \mathbf{x})$$

where $\mathbf{v}_W$ denotes the vectorization of $\mathbf{W}$. The Jacobian of the overall neural-synaptic system is then:

$$\mathbf{J} = \begin{bmatrix} \frac{\partial \mathbf{f}}{\partial \mathbf{x}} & \frac{\partial \mathbf{f}}{\partial \mathbf{v_W}} \\ \frac{\partial \mathbf{g}}{\partial \mathbf{x}} & \frac{\partial \mathbf{g}}{\partial \mathbf{v_W}} \end{bmatrix} \tag{3.1.1}$$

The goal will be to show that the off-diagonal blocks of this overall Jacobian cancel out in some metric, while the diagonal blocks are negative definite.

3.1.1 Off-Diagonal Blocks of the Jacobian

To begin, first consider the following observation. For weight dynamcics given by:

$$\forall i \neq j \;\; \dot{w}_{ij} = -x_i x_j - \gamma(t) w_{ij} \;\text{ and }\; \dot{w}_{ii} = -\frac{1}{2}(x_i)^2 - \gamma(t) w_{ii} \tag{3.1.2}$$

with $\gamma(t) > 0$, we have that the off-diagonal blocks of the overall Jacobian cancel out.

First we will show that w_{ij} converges to w_{ji} after exponential transients for (3.1.2). Consider a simple distance function between w_{ij} and w_{ji}:

$$V = (w_{ij} - w_{ji})^2$$

evaluating the time-derivative of V and substituting in (3.1.2), we see that

$$\dot{V} = 2(w_{ij} - w_{ji})(-x_i x_j - \gamma(t) w_{ij} + x_i x_j + \gamma(t) w_{ji}) = -2\gamma(t)V$$

which implies that the distance between w_{ij} and w_{ji} shrinks exponentially with at least rate $\min_t(\gamma(t))$, independently of initial condition or the particular trajectory of $\mathbf{x}$. For the rest of the analysis we will therefore assume that $w_{ij} \approx w_{ji}$. Using the symmetry of $\mathbf{W}$, the following four relationship can be shown:

$$\forall i \neq j \;\; \frac{\partial \dot{w}_{ij}}{\partial x_i} = -x_j \;\text{ and that }\; \frac{\partial \dot{x}_i}{\partial w_{ij}} = x_j$$

as well as

$$\forall i \neq j \;\; \frac{\partial \dot{w}_{ji}}{\partial x_i} = -x_j \;\text{ and that }\; \frac{\partial \dot{x}_i}{\partial w_{ji}} = x_j$$

The first three of these relationships can be verified by direct differentiation of (3.1.2). The last relationship can be seen by using the symmetry of $\mathbf{W}$. In particular, that:

$$\frac{\partial \dot{x}_i}{\partial w_{ji}} \approx \frac{\partial \dot{x}_i}{\partial w_{ij}} = x_j$$

What we have shown now is that under the synaptic dynamics defined above, we have the following relationship:

$$\frac{\partial \mathbf{g}}{\partial \mathbf{x}} = -\frac{\partial \mathbf{f}}{\partial \mathbf{v}_W}^T \tag{3.1.3}$$

Now , define a 'Hebbian' term as $\mathbf{H} \equiv \mathbf{x}\mathbf{x}^T$. The synaptic dynamics defined by (3.1.2) can in matrix notation can be written as

$$\dot{\mathbf{W}} = -\mathbf{P} \odot \mathbf{H} - \gamma(t)\mathbf{W} \tag{3.1.4}$$

where

$$P_{ij} = \begin{cases} 1 & \text{if } i \neq j \\ \frac{1}{2} & \text{if } i = j \end{cases}$$

vectorizing (3.1.4) and using the relation (1.1.11), we can see that

$$\dot{\mathbf{v}}_W = -\mathbf{D}_P \mathbf{v}_H - \gamma(t)\mathbf{v}_W$$

and differentiating this expression with respect to $\mathbf{x}$, we have:

$$\frac{\partial \dot{\mathbf{v}}_W}{\partial \mathbf{x}} = -\mathbf{D}_P \frac{\partial \mathbf{v}_H}{\partial \mathbf{x}} = -\frac{\partial \mathbf{f}}{\partial \mathbf{v}_W}$$

and finally, left multiplying this expression by the inverse of $\mathbf{D}_P$ we have

$$\frac{\partial \mathbf{v}_H}{\partial \mathbf{x}} = \mathbf{D}_P^{-1} \frac{\partial \mathbf{f}}{\partial \mathbf{w}}^T \tag{3.1.5}$$

Note that this expression is only true when $\mathbf{W}$ is symmetric. Now consider the synaptic dynamics in the main text:

$$\dot{\mathbf{W}} = -\mathbf{K} \odot \mathbf{H} - \gamma(t)\mathbf{W}$$

where $\mathbf{K}$ is symmetric , positive semi-definite and has positive entries. Vectorizing in the same manner as above and substituting in (3.1.5), we find that

$$\frac{\partial \mathbf{v}_{\dot{W}}}{\partial \mathbf{x}} = -\mathbf{D}_K \frac{\partial \mathbf{v}_H}{\partial \mathbf{x}} = -\mathbf{D}_K \mathbf{D}_P^{-1} \frac{\partial \mathbf{f}}{\partial \mathbf{v}_W}$$

If $\mathbf{D}_K$ is positive (which will happen when $\mathbf{K}$ has all positive entries), then $\mathbf{D}_K \mathbf{D}_P^{-1}$ is diagonal and positive definite, and thus Theorem 1 applies–this is a feedback combination that automatically preserves contraction.In other words, a metric can be found in terms of $\mathbf{D}_K \mathbf{D}_P^{-1}$ which leaves the diagonal blocks of the Jacobian untouched and forces the off-diagonal blocks to cancel out when computing the symmetric part of the Jacobian.

3.1.2 Diagonal Blocks of the Jacobian

The upper left block of the Jacobian is

$$\frac{\partial \mathbf{f}}{\partial \mathbf{x}} = \frac{\partial \mathbf{h}}{\partial \mathbf{x}} + \mathbf{W}(t)$$

thus, for this matrix to be negative definite we need

$$\mu_2(\mathbf{W}(t)) < \beta \tag{3.1.6}$$

To see that this will be the case, consider that the synaptic dynamics defined in the main text can be written in matrix notation as

$$\dot{\mathbf{W}} = -\mathbf{K} \odot \mathbf{x}\mathbf{x}^T - \gamma(t)\mathbf{W}$$

Now discretize the differential equations for $\mathbf{W}$:

$$\dot{\mathbf{W}} \approx \frac{\mathbf{W}_{t+1} - \mathbf{W}_t}{\Delta t} = -\mathbf{K} \odot \mathbf{x}_t \mathbf{x}_t^T - \mathbf{W}_t$$

rearranging this to collect all the t terms on the right hand side, we have

$$\mathbf{W}_{t+1} = (1 - \Delta t)\mathbf{W}_t - \Delta t\mathbf{K} \odot \mathbf{x}_t\mathbf{x}_t^T$$

To make derivations less tedious, let's define $\beta \equiv \Delta t > 0$ and $\alpha \equiv (1 - \beta)$. Note that since that, ideally, Δt is small, we can say that $0 < \alpha < 1$. Now we have the discrete dynamical system for $\mathbf{W}$:

$$\mathbf{W}_{t+1} = \alpha\mathbf{W}_t - \beta\mathbf{K} \odot \mathbf{x}_t\mathbf{x}_t^T$$

We know need to notice that $-\mathbf{K} \odot \mathbf{x}_t\mathbf{x}_t^T$ is symmetric (since $\mathbf{K}$ and $\mathbf{x}_t\mathbf{x}_t^T$ are symmetric). It is also negative semi-definite, by the Schur Product Theorem. Let's denote this expression by $\mathbf{N}_t = \mathbf{N}_t^T \prec 0$ so that

$$\mathbf{W}_{t+1} = \alpha\mathbf{W}_t + \beta\mathbf{N}_t$$

Taking the symmetric part of both sides we find that:

$$(\mathbf{W}_{t+1})_s = \alpha(\mathbf{W}_t)_s + \beta\mathbf{N}_t$$

We can now use Weyl's inequality for the sum of Hermitian matrices (see premilinaries) to conclude that

$$\mu_2(\mathbf{W}_{t+1}) \leq \alpha\mu_2(\mathbf{W}_t)$$

and therefore that

$$\mu_2(\mathbf{W}_t)) \leq \alpha^t\mu_2(\mathbf{W}_0)$$

which, since $0 < \alpha < 1$, means that the symmetric part of $\mathbf{W}$ decays exponentially to zero. Recall that for contraction we needed $\mu_2(\mathbf{W}) < \beta$. Assuming that $\mu_2(\mathbf{W}_0) > \beta$, the upper-bound for the number of steps this will take is $O(\ln(\frac{\mu_2(\mathbf{W}_0)}{\beta}))$.

To summarize, we have shown that the synaptic dynamics described by

$$\dot{\mathbf{W}} = -\mathbf{K} \odot \mathbf{x}\mathbf{x}^T - \gamma(t)\mathbf{W} \tag{3.1.7}$$

where $\mathbf{K}$ is a symmetric, positive semi-definite matrix with positive entries, lead to contracting neural and synaptic dynamics in a wide class of RNNs. We did this by showing that the overall Jacobian (3.1.1) has diagonal blocks that 'cancel' out and diagonal blocks that are negative definite–implying negativedefinitness of the whole Jacobian and thus contraction of the combined neural and synaptic system. Crucially, to show this we had to use a metric other than the identity metric.

4 Sparsity Ensures Contraction

In this section we show that it is possible to ensure contraction–without any knowledge of the specific synaptic dynamics–by keeping the network sufficiently sparse. This result is a straightfoward application of the μ_∞ matrix measure to the Jacobian of the overall neural and synaptic system. Consider neural networks of the form

$$\dot{x}_i = h(x_i) + \sum_{j \neq i}^{N} W_{ij} r_j + u_i(t) \tag{4.0.1}$$

where h_i is a leak-term and r_i is bounded activation function that satisfies the following two properties:

$$|r_i| \leq r_{max} \ \text{ and } \ 0 < g_{min} \leq \frac{\partial r_i}{\partial x_i} \leq g_{max} \tag{4.0.2}$$

We consider synaptic dynamics of the form

$$\dot{W}_{ij} = g(W_{ij}, x_i, x_j) \tag{4.0.3}$$

That is to say, we only consider synaptic dynamics that are *local*, in the sense that the change in weight is only a function of the weight itself, and the pre and post synaptic neurons.

It will be useful to define, for neuron i, the total number of afferent synapses and the total number of afferent *dynamic* synapses which we denote p_i and d_i, respectively. Note that d_i is a fraction of p_i and can therefore be written as

$d_i = \alpha_i p_i$ for some $0 \leq \alpha_i \leq 1$. It will also be useful to give names to one more global system parameter: the maximum amplitude (absolute value) of all the weights in the network: $w_* \equiv \max_{ij}(|w_{ij}|)$. For the neural rows of the Jacobian, we see that the μ_∞ measure can be upper bounded as

$$sup_i(\frac{\partial h_i}{\partial x_i} + \sum_{j \neq i}^{N} |\frac{\partial \dot{x}_i}{\partial x_i}| + \sum_{q=1}^{d_i} |\frac{\partial \dot{x}_i}{\partial W_{iq}}|) < 0 \tag{4.0.4}$$

By using the following two relations:

$$\frac{\partial \dot{x}_i}{\partial x_j} = W_{ij} \frac{\partial r_j}{\partial x_j} \text{ and } \frac{\partial \dot{x}_i}{\partial W_{ij}} = r_j$$

we can see that (4.0.4) can be assured if

$$\forall i, t \;\; p_i g_{max} w_* + d_i r_{max} < \beta$$

Substituting in the relationship $d_i = \alpha_i p_i$ into the above, we get:

$$\forall i, t \;\; p_i [g_{max} w_* + \alpha_i r_{max}] < \beta \tag{4.0.5}$$

We still have the 'synaptic' rows of the Jacobian to worry about, but these are much simpler. The locality assumption implies that there are *at most* three elements per row. These elements are the derivatives of g_{ij} with respect to x_i and x_j and the derivative of g_{ij} with respect to w_{ij}. Thus, if

$$\forall i, j, t \;\; |\frac{\partial g_{ij}}{\partial x_i}| + |\frac{\partial g_{ij}}{\partial x_j}| + \frac{\partial g_{ij}}{\partial w_{ij}} < 0 \tag{4.0.6}$$

the bottom two Jacobian blocks satisfy the matrix measure condition. Intuitively, this inequality tells us that the 'self-stabilization' of the synapses must be stronger than the change in plasticity due to neural activity. For example, if one imagines that g_{ij} contains a constant leak term of the form $-\gamma w_{ij}$ where $\gamma > 0$, then the inequality above is a sufficient condition on how large γ should be.

In summary, we have shown that if the synapses are contracting (i.e (4.0.6) is satisfied) then (4.0.5) tells us that it is possible to assure contraction of the overall neural and synaptic network by making the network sufficiently sparse.

5 Contraction in RNNs with Static Weights

In this section we'll analyze RNNs of the general form

$$\dot{x}_i = h_i(x_i) + \sum_{i=1}^{n} W_{ij}\phi(g_i x_i) + u_i(t) \tag{5.0.1}$$

Where h_i is a leak-term (see (2.2.4) for definition), ϕ is a nonlinear function with a linear 'regime' (such as tanh(x)), g_i is a neuron-specific gain-term that satisfies:

$$\forall i, \;\; 0 < g_{min} \leq g_i \leq g_{max}$$

and $u_i(t)$ is a time-varying input into neuron i. For simplicity, we focus here on the regime where

$$\phi(g_i x_i) \approx g_i x_i$$

In this case we may rewrite (5.0.1) in matrix form as:

$$\dot{\mathbf{x}} = \mathbf{h}(\mathbf{x}) + \mathbf{W}\mathbf{G}\mathbf{x} + \mathbf{u}(t) \tag{5.0.2}$$

where $\mathbf{G}$ is a constant diagonal, positive definite matrix such that $G_{ii} = g_i$. The Jacobian of (5.0.1) is a state-dependent matrix:

$$\mathbf{J} = \mathbf{L} + \mathbf{W}\mathbf{G} \tag{5.0.3}$$

where $\mathbf{L}$ is a diagonal matrix such that $L_{ii} = \frac{\partial h_i}{\partial x_i} \leq -\beta$. Consider the following metric transformation to yield a generalized Jacobian, $\mathbf{F}$:

$$\mathbf{\Theta} = \mathbf{G}^{1/2}\mathbf{D} \tag{5.0.4}$$

Where $\mathbf{D}$ is an arbitrary, non-singular diagonal matrix. Applying this metric to (5.0.3) we find that

$$\begin{aligned}\mathbf{F} &= \mathbf{\Theta J \Theta}^{-1} \\ &= \mathbf{\Theta L \Theta}^{-1} + \mathbf{\Theta W G \Theta}^{-1} \\ &= \mathbf{L} + \mathbf{G}^{1/2}[\mathbf{D W D}^{-1}]\mathbf{G}^{1/2} \\ &= \mathbf{G}^{1/2}[\mathbf{G}^{-1}\mathbf{L} + \mathbf{D W D}^{-1}]\mathbf{G}^{1/2}\end{aligned} \tag{5.0.5}$$

Which implies that a sufficient condition for contraction in the RNN described by (5.0.2) is that

$$\mu_{D,2}(\mathbf{W}) + \frac{\beta}{g_{max}} \leq -c, \quad c > 0 \tag{5.0.6}$$

If (5.0.6) is true, then the network described by (5.0.2) is contracting with rate of $\lambda_F = c\, g_{min}$.

5.0.1 Contraction in Circuits with Perfect E-I Balance

One practical implication of (5.0.6) is that we can now exploit feedback structure present in $\mathbf{W}$ in a way that would have been missed by using the spectral norm. As an example, consider a network with weight matrix

$$\mathbf{W} = \begin{bmatrix} \mathbf{W}_{EE} & \mathbf{W}_{EI} \\ \mathbf{W}_{IE} & \mathbf{W}_{II} \end{bmatrix} \tag{5.0.7}$$

Assume that $\mathbf{W}_{IE}$ is a matrix containing all positive entries and $\mathbf{W}_{EI}$ is a matrix containing all negative entries. Further assume that:

$$-k\mathbf{W}_{IE}^T = \mathbf{W}_{EI} \tag{5.0.8}$$

where $k > 0$. In other words, (5.0.8) asserts that this circuit has perfect E-I balance, up to a constant scalar factor. In this case, the weight matrix becomes:

$$\mathbf{W} = \begin{bmatrix} \mathbf{W}_{EE} & -k\mathbf{W}_{IE}^T \\ \mathbf{W}_{IE} & \mathbf{W}_{II} \end{bmatrix}$$

This corresponds to two interacting neural populations, one inhibitory and one excitatory. The excitatory population excites the inhibitory one through the $\mathbf{W}_{IE}$ submatrix and the inhibitory population inhibits the excitatory population through the $-k\mathbf{W}_{IE}^T$ submatrix. Denote the (unweighted) matrix measures of the EE and II subnetworks μ_{EE} and μ_{II}, respectively:

$$\mu_2(\mathbf{W}_{EE}) = \mu_{EE} \ \text{ and } \ \mu_2(\mathbf{W}_{II}) = \mu_{II} \tag{5.0.9}$$

It's straightforward to show that by picking $\mathbf{D}$ as:

$$\mathbf{D} = \begin{bmatrix} \mathbf{I} & 0 \\ 0 & \sqrt{k}\mathbf{I} \end{bmatrix}$$

we get:

$$\begin{aligned} \mu_{D,2}(\mathbf{W}) &= \frac{1}{2}\lambda_{\max}([\mathbf{D}\mathbf{W}\mathbf{D}^{-1} + \mathbf{D}^{-1}\mathbf{W}^T\mathbf{D}]) \\ &= \frac{1}{2}\lambda_{\max}(\begin{bmatrix} \mathbf{W}_{EE} + \mathbf{W}_{EE}^T & 0 \\ 0 & \mathbf{W}_{II} + \mathbf{W}_{II}^T \end{bmatrix}) \\ &= \max[\mu_2(\mathbf{W}_{EE}), \mu_2(\mathbf{W}_{II})] \end{aligned} \tag{5.0.10}$$

which implies that

$$\mu_{D,2}(\mathbf{W}) = \max[\mu_{EE}, \mu_{II}]$$

This shows that by appropriately scaling μ_{EE} and μ_{II} to satisfy (5.0.6), the RNN can be made contracting *independent* of the strength of individual elements in the diagonal blocks.

5.0.2 Contraction in Circuits With Statistical E-I Balance

In a biological system, perfect E-I balance (i.e (5.0.8)) cannot reasonably be expected. However, some basic results from matrix algebra and random matrix theory can tell us that contraction can be assured even in the case where E-I balance occurs in an average sense.

In particular, we'll need the following result from matrix algebra. Consider a symmetric block matrix of the form:

$$\begin{bmatrix} \mathbf{A} & \mathbf{B} \\ \mathbf{B}^T & \mathbf{C} \end{bmatrix} \tag{5.0.11}$$

where $\mathbf{A}$ and $\mathbf{C}$ are negative definite and symmetric, with $\lambda_{\max}(\mathbf{A}) = -\lambda_A < 0$ and $\lambda_{\max}(\mathbf{C}) = -\lambda_C < 0$. Then a standard result from matrix algebra [3] says that a sufficient condition for negative-definiteness of (5.0.11) is that:

$$||\mathbf{B}||_2^2 < \lambda_A \lambda_C \tag{5.0.12}$$

We'll also need the following result from random matrix theory. Consider an $N \times N$ symmetric matrix, $\mathbf{R}$, whose elements above and including the diagonal are distributed according to a Gaussian distribution:

$$R_{ij} = \mathcal{N}(0, \sigma^2) \;\text{ for }\; j \geq i$$

Then for large N, we have from [2] that, in expectation:

$$||\mathbf{R}||_2 \approx 2\sigma\sqrt{N} \tag{5.0.13}$$

We can use (5.0.12) together with (5.0.13) for how much deviation from perfect E-I balance a circuit of size N can tolerate while preserving contraction. In particular, consider again the matrix defined in (5.0.7) and assume that (5.0.6) is satisfied for each diagonal block. That is, assume:

$$\mu_{EE} + \beta g_{max}^{-1} \leq -c \;\text{ and }\; \mu_{EE} + \beta g_{max}^{-1} \leq -c \;\text{ with }\; c > 0$$

and assume $N_E = N_I = \frac{N}{2}$ for simplicity. We define $\mathbf{R}$ as follows:

$$\mathbf{R} \equiv \frac{\mathbf{W}_{EI} + \mathbf{W}_{IE}^T}{2}$$

from (5.0.12), we can assure contraction in regions where:

$$||\mathbf{R}||_2 < c$$

To find this region, we will upper bound the spectral norm of $\mathbf{R}$ as follows: instead of the case where E-I and I-E connections cancel out exactly, consider the case when instead they are each drawn from a distribution whose *means* cancel out exactly. Namely:

$$W_{IE}^{ij} \sim \mathcal{N}(m, \sigma_{IE}^2) \;\text{ and }\; W_{EI}^{ij} \sim \mathcal{N}(-m, \sigma_{EI}^2)$$

This implies that:

$$R_{ij} = \frac{W_{IE}^{ij} + W_{EI}^{ji}}{2} \sim \mathcal{N}(0, \frac{\sigma_{IE}^2 + \sigma_{EI}^2}{4})$$

Thus, the matrix $\mathbf{R}$ is a symmetric, Gaussian matrix with zero mean and variance $\frac{\sigma_{IE}^2+\sigma_{EI}^2}{4}$. By plugging this expression into (5.0.13) we can conclude contraction when the following relationship holds between $\mathbf{R}$ and the eigenvalues of the E-E and I-I connection matrices:

$$\sigma_{IE}^2 + \sigma_{EI}^2 < \frac{c^2}{N}$$

Of course, in the limit as the spreads go to zero, we arrive back at the case of perfect E-I balance with $k = 1$, which always yields contraction.

Another way to get a sense of the tolerance' for imperfect E-I balance is to use the Frobenius norm of $\mathbf{R}$, which upper-bounds the spectral norm. Assume that for each connection between excitatory neuron i and inhibitory neuron j, we have that

$$|W_{EI}^{ij} + W_{IE}^{ji}|^2 \leq \kappa^2 \;\text{ with }\; \kappa > 0$$

This straightforwardly implies that the Frobenius norm of $\mathbf{R}$ is upper bounded by:

$$||\mathbf{R}||_F \leq \kappa N$$

Which means that it is possible to ensure contraction by scaling κ with N, since:

$$||\mathbf{R}||_2 \leq ||\mathbf{R}||_F \leq \kappa N < c$$

So by scaling κ, which measures how imperfectly each E-I synapse is balanced by the corresponding I-E synapse, we can guarantee contraction.

5.1 Relationship to Echo State Networks

Echo state networks (ESNs) are contracting, nonlinear, discrete-time RNNs with static synaptic weights. While attempts have been made to apply the Echo-State framework to continuous time systems, these attempts have relied on discretizations of the underlying continuous dynamics. What determines if a network is an ESN is the 'Echo-State Property' (ESP), which is typically in the form of a bound on the spectral norm of the weight matrix. Unlike our result above (see 5.0.6) this does not allow one to factor in stabilizing influences within the weight matrix, such as E-I balance. Nevertheless, contraction analysis can be applied to discrete time systems. For completeness, we will now do this to and derive a slight generalization of a known sufficient condition for the ESP in networks where the activation function is more flexible than $\tanh(x)$.

We will need the following result. Consider a discrete-time dynamical system:

$$\mathbf{x}_{n+1} = \mathbf{T}(\mathbf{x}_n, \mathbf{u}_n)$$

It was shown in [4] that this system will be contracting if there exists some non-singular matrix $\mathbf{\Theta}_n$ such that

$$\sigma_{\max}(\mathbf{\Theta}_{n+1}\frac{\partial \mathbf{T}}{\partial \mathbf{x}}\mathbf{\Theta}_n^{-1}) < 1$$

Theorem 2. *Consider the discrete time RNN*

$$\mathbf{x}_{n+1} = \mathbf{W}\mathbf{r}(\mathbf{x}_n + \mathbf{u}_n) \tag{5.1.1}$$

where $r_i = r_j = r$ is a nonlinearity (e.g. tanh(x)) that satisfies

$$0 \leq \frac{\partial r}{\partial x}(x) \leq g \tag{5.1.2}$$

If the matrix $\tilde{\mathbf{W}} \equiv \sqrt{g}\mathbf{W}$ *is Schur diagonally stable, then the RNN is contracting, and therefore has the ESP. That is, if there exists a postive definite, diagonal matrix* $\mathbf{P}$ *such that:*

$$\tilde{\mathbf{W}}^T\mathbf{P}\tilde{\mathbf{W}} - \mathbf{P} < 0 \ \ \textit{with} \ \ \tilde{\mathbf{W}} \equiv g\mathbf{W}$$

the RNN is contracting.

Proof. For the RNN defined in the theorem, we have

$$\sigma_{\max}(\mathbf{\Theta}_{n+1}\mathbf{W}\mathbf{\Phi}\mathbf{\Theta}_n^{-1}) < 1$$

Let $\Theta_n = \mathbf{P}^{1/2}$, where $\mathbf{P}$ is diagonal and positive definite. Using the submultiplicitivity of the spectral norm, we find that a sufficient condition for contraction is:

$$\sigma_{\max}(\mathbf{P}^{1/2}\mathbf{W}\mathbf{P}^{-1/2}) < g^{-1}$$

we can easily rewrite this condition as

$$\mathbf{W}^T\mathbf{P}\mathbf{W} - \mathbf{P}g^{-2} < 0$$

Multiplying both sides by g^2, we find that the RNN is contracting if

$$g^2\mathbf{W}^T\mathbf{P}\mathbf{W} - \mathbf{P} < 0 \iff \tilde{\mathbf{W}}^T\mathbf{P}\tilde{\mathbf{W}} - \mathbf{P} < 0$$

Which can be readily recognized as the statement that the matrix $\tilde{\mathbf{W}} = g\mathbf{W}$ is Schur diagonally stable. □

Remark 1. *The above stability criterion immediatly applies to RNNs of the form:*

$$\mathbf{x}_{n+1} = \mathbf{r}(\mathbf{W}\mathbf{x}_n + \mathbf{u}_n)$$

for nonsingular $\mathbf{W}$*. This can be seen using the following coordinate transform and applying it to* (5.1.1)*:*

$$\mathbf{y} = \mathbf{W}^{-1}\mathbf{x}$$

$$\mathbf{y}_{n+1} = \mathbf{W}^{-1}\mathbf{x}_{n+1} = \mathbf{W}^{-1}[\mathbf{W}\mathbf{r}(\mathbf{x}_n + \mathbf{u}_n)] = \mathbf{r}(\mathbf{W}\mathbf{y}_n + \mathbf{u}_n)$$

Since $\delta\mathbf{y} = \mathbf{W}^{-1}\delta\mathbf{x}$*, this means that* $\delta\mathbf{x} \to 0 \iff \delta\mathbf{y} \to 0$*,*

Remark 2. *For the specific case of* $r(x) = \tanh(x)$*,and therefore* $g = 1$*, this result already appears in [8].*

Remark 3. *A common RNN uses the discontinuous activation function:*

$$\mathbf{r}(x_i) = \max(0, x_i)$$

This does not pose a problem for the above stability condition, for the following reason. Define a diagonal, 'switching' matrix $\mathbf{S}$ *in the following way:*

$$S_{ii} = \begin{cases} 1 & \textit{if } x_i > 0 \\ 0 & \textit{otherwise} \end{cases}$$

And note that the dynamics of the ReLu network can be written:

$$\mathbf{x}_{n+1} = \mathbf{W}\mathbf{S}\mathbf{x}_n + \mathbf{u}_n$$

The Jacobian of this system is:

$$\mathbf{J} = \mathbf{W}\mathbf{S}$$

Since $\mathbf{S}$ *is diagonal and* $||\mathbf{S}|| \leq 1$*, we can use the same diagonal metric transformation as above to find that a sufficient condition for a ReLu RNN to be contracting is that* $\mathbf{W}$ *is Schur diagonally stable. In other words, if there exists some positive and diagonal* $\mathbf{P}$ *such that*

$$\mathbf{W}^T\mathbf{P}\mathbf{W} - \mathbf{P} < 0$$

the ReLu network is contracting.

References

[1] Desoer, C., and Haneda, H. The measure of a matrix as a tool to analyze computer algorithms for circuit analysis. *IEEE Transactions on Circuit Theory 19*, 5 (1972), 480–486.

[2] Füredi, Z., and Komlós, J. The eigenvalues of random symmetric matrices. *Combinatorica 1*, 3 (1981), 233–241.

[3] Horn, R. A., Horn, R. A., and Johnson, C. R. *Matrix analysis.* Cambridge university press, 1990.

[4] Lohmiller, W., and Slotine, J.-J. E. On contraction analysis for nonlinear systems. *Automatica 34*, 6 (1998), 683–696.

[5] Slotine, J.-J. E., Li, W., et al. *Applied nonlinear control*, vol. 199. Prentice hall Englewood Cliffs, NJ, 1991.

[6] Sontag, E. D. Contractive systems with inputs. In *Perspectives in Mathematical System Theory, Control, and Signal Processing.* Springer, 2010, pp. 217–228.

[7] Vidyasagar, M. *Nonlinear systems analysis*, vol. 42. Siam, 2002.

[8] Yildiz, I. B., Jaeger, H., and Kiebel, S. J. Re-visiting the echo state property. *Neural networks 35* (2012), 1–9.
