# Supplementary material for: Achieving stable dynamics in neural circuits
Source: PLoS Comput Biol. 2020 Aug 7;16(8):e1007659. doi: 10.1371/journal.pcbi.1007659 (PMC7446801; doi:10.1371/journal.pcbi.1007659)
Supplement: Attachment — Submitted filename: StableDynamicsPLOSReviewResponses3.docx [file pcbi.1007659.s002.docx]

Reviewer #1: The authors have some interesting results that would be useful for theorists/modelers in computational neuroscience to be aware of. The usefulness would be enhanced if some extra work is done to clarify the findings and state the limits on their generality or whether they could be expanded. I point out the areas where this can be achieved below:

**Our reply:**

**We thank the reviewer for their kind words and support.**

l.57 “all such trajectories converge”: this suggests the focus is on networks with a single fixed point. Are these really the interesting ones? Is there some generalization to networks with multiple fixed points where one can discuss regions of contraction? Such networks may be more versatile and useful.

**Our reply:**

**We thank the reviewer for pointing out this ambiguity in our writing. We have edited the abstract and the introduction to reflect the following: it is correct that *in the absence of external input*, globally contracting systems settle down to a single fixed point. However, when a time-varying input is presented to a contracting system, the resulting trajectories of the system are generally time-varying (as can be seen in Fig 2, main text). This gives contracting neural networks more versatility than neural networks that only converge to a single fixed point.**

Eq. 1: The description “in the absence of input from other neurons” suggests that the diagonal part of the connection matrix, W_ij is contained within the first term, h(x). Is this true? Or are you only considering W_ij without diagonal self-connection terms? If not, then “other” neurons should be modified to include self-feedback.

**Our reply:**

**The reviewer is correct that this is an ambiguous definition. In our networks, h(x) does not include any self-connection terms. Self-connection terms are introduced through the diagonal elements of the weight matrix, as the reviewer astutely points out. We have edited the manuscript to more explicitly define h(x).**

Eq. 1: It is important to indicate the range of “x” as it appears to enter the expression as if it is a firing rate which can only be non-negative. Are you allowing negative neural outputs?

Eq.2: Following the above comment, if “x” represents firing rate so can only be non-negative, then the equation for changes in connection strengths prevent any stable positive connections from arising (the initial term is negative or zero and the second term has opposite sign to W). This seems wrong. Similarly if x can be negative some discussion should be made about the meaning of negative rates and how the change in synaptic strength should depend on such rates. Perhaps zero “x” corresponds to a threshold for the rate-dependent switch between synaptic depression and potentiation and the formulae would all work with shifted “x” and threshold subtracted, to represent non-negative firing rates?

l. 130: I think the logic is correct but the explanation a little too brief:
According to Hebb’s rule if a neuron helps cause another neuron to fire then a change in connection enhances the likelihood of such a causal event occurring again. i.e. Hebb’s rule is all about positive feedback (the synapse changes to enhance existing correlations).
For excitatory neurons that means strengthening following correlated spikes.
For an inhibitory neuron, it can help a downstream neuron fire by not spiking at the same time.
Also, a decrease in the inhibitory synapse will help the postsynaptic neuron fire.
So, firing at different times (anticorrelated) should lead to a decrease in inhibitory strength (Wij more positive) as it is then more likely that the inhibitory cell helps the excitatory cell fire -- or conversely, firing at the same time leads to an increase in inhibitory strength (Wij more negative as stated here). Perhaps a short-hand could be that a “positive feedback” process is Hebbian and a negative feedback process is anti-Hebbian? However, I am still confused by the values of “x” and their sign being perhaps negative. It may help to describe this as negative x meaning low-rate and positive x meaning high-rate. But then this rule would allow for strong plasticity when both pre- and post-synaptic rates are minimal (x very negative) in contradiction to known plasticity mechanisms. These issues should be discussed.

**Our reply:**

**We agree that we did not fully discuss the interpretation of equation (1). We have chosen to reply to all three of the above comments at the same time because they all stem from the same issue—our lack of discussion regarding the interpretation of equation (1). We agree with the reviewer that this discussion is indeed critical to the interpretation of the results as a whole, so we have expanded section 3.1 to include an interpretation of equation (1).**

**To summarize briefly here, we follow previous authors in interpreting *x* as a deviation from a baseline, spontaneous firing rate. This carries a main unstated assumption, which is that the baseline firing rates are positive– thus allowing for *x* to be negative—and large enough so that *baseline + x > 0.* Since contracting systems are bounded, it is always possible to find such a baseline and so this does not change the mathematical substance of our results. However, it is indeed important to state this assumption clearly (which we have done in the main text directly under equation 1).**

**We also agree with the reviewer that more discussion is also needed with regards to the interpretation of equation (2). Given this interpretation of equation (1), the interpretation of equation (2) becomes hopefully clearer. Nevertheless, we agree with the reviewer that a more detailed discussion of equation (2) is also needed. Thus we have edited the main text to include a detailed discussion of equation (2) as well as a summarizing table (Table 1 in main text) for convenience. To briefly reiterate that discussion here: following other authors, by Hebbian plasticity we refer to the increase of synaptic efficiency under correlated pre and post synaptic neural activity. For the neural networks we consider (which have scalar weights) synaptic efficiency is measured by** $\boldsymbol{|}\boldsymbol{w}_{\boldsymbol{ij}}\boldsymbol{|}$**.**

**For an inhibitory synapse (**$\boldsymbol{w}_{\boldsymbol{ij}}\boldsymbol{<0)}$ **) if both neuron**$\boldsymbol{j}$ **and neuron** $\boldsymbol{i}$ **have correlated activity, meaning that their firing rates either decrease or increase from their respective baselines in the same time window, then equation (2) implies that** $\boldsymbol{w}_{\boldsymbol{ij}}$ **should *decrease.* However, since** $\boldsymbol{w}_{\boldsymbol{ij}}$ **is negative, this implies that the efficiency of the synapse, captured by** $\boldsymbol{|}\boldsymbol{w}_{\boldsymbol{ij}}\boldsymbol{|}$ **will *increase*—implying that for these inhibitory synapses, equation (2) describes Hebbian plasticity. For an excitatory synapse (**$\boldsymbol{w}_{\boldsymbol{ij}}\boldsymbol{>0)}$ **) the opposite is true: the efficiency of the synapse will decrease under correlated neural activity—implying that for excitatory synapses, equation (2) describes what is commonly referred to as anti-Hebbian plasticity.**

Figure 3: Please define “Norm of Matrix Component” via an equation
l. 146 typo (“of”)

**Our reply:**

**We thank the reviewer for spotting this typo, we’ve corrected it in the main text.**

l. 173-174 The calculation is incorrect. You are comparing connections per neuron with total connections in the brain. The numerator should be multiplied by number of neurons (10^11).

**Our reply:**

**We thank the reviewer for spotting this embarrassing error. We’ve corrected it in the main text.**

l. 197: It seems that there are two separate (but linked) systems operating on very different time scales. On the fast time scale, for a given weight matrix, one can ask if the neural firing rates of the system are contracting. At times (especially when Echo State networks are compared or the requirements for weight matrices) it seems like the goal is to generate contracting networks in terms of neural firing rates. However, l. 197 suggests that the discussions of contraction are in the components of the weight matrix. The authors should clearly separate discussions and results concerning weight matrices that contract (such that the network produces a reliable/reproducible – perhaps in this case temporally variable – set of synaptic strengths) versus whether the firing rates of neurons follow relatively stable trajectories.

**Our reply:**

**We thank the reviewer for raising this point and highlighting that we did not state the goals of each section explicitly enough. We correct this in the main text by adding a sentence at the end of the introduction. The sections 3.1 and 3.2 are indeed concerned with contraction of *both* the neural activity and the components of the weight matrix (as can be seen in Fig 1,2 & 3) while the remaining sections are concerned solely with the contraction of neural activity, since the weights are held fixed (i.e are parameters, not variables).**

Eq. 4: The terms here need a little more description and explanation of how they enter. While the main point is trivial (decreasing p can lead to stability) the terms like “g_max” appear to be related to the maximum derivative of connection strength with respect to neural activation (Eq. 4.0.3) rather than what is generally termed “gain” of a neuron (gradient of f-I curve). Also, does w_max refer to maximum strength of plastic synapses (in which case why is it not in the “alpha” term) or is it simply the maximum value of the assigned fixed synapses? Perhaps explain why increasing the fraction of static to plastic synapses allows for a larger w_max.

**Our reply:**

**This is a very helpful suggestion. We expanded this section in the main text to define mathematically and discuss the meaning of each variable in equation 4. The term g_max is indeed related to the gradient of the f-I curve—it is simply the maximum of this gradient. W_max refers to the maximum strength of any synapse, including dynamic ones. The bound in (4) is achieved by summing over the absolute value of all the elements in the ith row of the overall Jacobian and seeing if it is less than the ith diagonal term of the overall Jacobian. Since increasing the fraction of static to plastic synapses decreases the number of elements of the Jacobian, there is more ‘room’ for W_max to grow while still satisfying the inequality in (4). We now point this out in the MS.**

l. 211: “has” not “have”

l. 232 type “that” should be “the”

Fig. 4: legend is pretty opaque and stops half-way through a sentence in my version.

l. 346 “increases” not “increase”

**Our reply:**

**We thank the reviewer for spotting these typos, we’ve corrected them in the main text.**

l. 218 “impact of such synapses cancel” but there are time-differences in practice that commonly lead to oscillations. I think “cancel” is an overstatement.

l.224-225 “will not interfere with stability”: in fact the antisymmetric part can enhance stability (this is easy to see from the determinant of a 2x2 matrix). So, while the intent is correct, the point could be made a little more clearly. That is, if the symmetric part is stable the complete matrix will be, however, if the symmetric part is not stable the antisymmetric part could be sufficient to generate stability overall.

l. 232 “the off-diagonal elements cancel out”. No! They impact the determinant of the matrix and hence can impact the values (and signs) of the eigenvalues, therefore impacting stability. But again, I think only in the sense of enhancing stability.

**Our reply:**

**The reviewer is correct that our language is incomplete here. We have edited the text to now say that the if E-I connections are balanced with I-E then they will not interfere *negatively* with stability. The reviewer is correct that there is still room for oscillatory behavior—the amplitude of this oscillatory behavior is simply upper bounded by a decaying exponential in the case of contraction. The reviewer is also absolutely correct in saying that the anti-symmetric parts of the Jacobian may in some cases interfere positively with stability—in other words, making the system *more* stable. We have changed our wording in the main text to clarify that the off-diagonal terms cancel in the sense of estimating ‘worst case’ bounds for stability, not that they cancel in the sense that they don’t impact dynamics at all.**

Section 3.4 on Echo State networks: this seems a little strange and overly expounded, especially when discussing the discrete-time nature of Echo State networks when there are almost identical Liquid State networks that gained prominence at the same time (sometimes in joint reviews) with the major difference being the Liquid State networks operate in continuous time. Why focus on Echo State not Liquid State given the latter is more similar to the current paper?

**Our Reply:**

**We agree that this point needs more discussing and refinement in the main text. We edited the main text section 3.4 to reflect this sentiment.**

**We also agree that very similar principles underlie both ESNs and LSMs. However, LSMs are not straightforwardly amenable to contraction analysis due to the discontinuous nature of the spiking ‘reset’ in the leaky-integrate and fire neurons they are typically implemented on. This spiking reset violates the smoothness and continuity assumptions needed to compute the Jacobian of the system, significantly complicating matters. We are, in fact, working on applying contraction analysis to these cases (of mixed continuous and discrete) neural dynamics. But we feel it is beyond the scope of the present work. We can promise the reviewer that this is being addressed in another manuscript.**

**Moreover, as ESNs are making a resurgence in the deep learning community, it is becoming clear that they are capable of performing complex and interesting neural computations. By highlighting the link between contraction and ESNs, we therefore demonstrate that contracting neural networks are in principle capable of performing useful and interesting neural computations. In other words, the strong stability properties of contracting neural networks do not automatically prohibit them from doing interesting computations.**

Discussion: I think papers that train some chaotic networks, for example Laje and Buonomano 2012, should be mentioned at a minimum and, even better, discussed in terms of contracting trajectories given that is what they appear to do. How is their plasticity rule or network similar or dissimilar to any mentioned in this paper that lead to contraction? Or is there system not contracting? Or only contracting in a portion of phase space? It would be nice to see this sort of analysis (even if only descriptive) applied to a few prior networks that appear to achieve contraction

**Our Reply:**

**We thank the reviewer for this helpful suggestion. We agree that the Discussion section should be edited to include discussion on Laje and Buonomano 2012. We have done this.**

**In the Laje and Buonomano paper, stability is experimentally observed, but not proven. This is the main difference of our approach: it provides provable certificates of stability for plastic RNNs. In some cases in the literature (for example Hennequin, 2014), linear stability around the origin is proven (which indeed implies that there is a contraction region around the origin) but the size of this region is neither established nor sought after. We expand on this in the main text and also added the Laje and Buonomano 2012 example to the Discussion, as we agree with the reviewer’s point.**

Reviewer #2:

Overview: The authors analyze the mechanisms that neural circuits employ in order to maintain stable dynamics in face of perturbations and plasticity through the lens of a control theoretic perspective, namely, contraction analysis. The study indicates that mechanisms such as inhibitory Hebbian plasticity, excitatory anti-Hebbian plasticity, synaptic sparsity and E-I balance can all lead to stability within the considered networks.
Comments:
• Contribution: The authors point out that stability of neural circuits has been considered in the literature widely, but what is less explored is how despite trial to trial variability, the brain manages to produce robust computations and therefore robust responses. The method considered here i.e., contraction analysis, states that the trajectories produced by population activity converge quickly following perturbation and therefore are robust in the face of small perturbations.

The paper brings together certain established analytical methods (contraction theory) to treat a long standing problem in computational neuroscience. While some of the factors discussed (inhibitory plasticity, E-I balance) have been studied in the context of achieving stability, they are brought together here under a common analytical framework. In this sense, the paper could constitute a useful contribution to the literature. However, the technical novelty is at time ambiguous and the presentation could be more precise in this regard. I elaborate on specific concerns below:

**Our Reply:**

**We thank the reviewer for their kind comments and support.**

• Main concerns.

o The authors frequently refer to computations in the Appendix section to narrate their findings. Important quantifications (not the extensive mathematical derivations) should be introduced in line with the claims made in the Result section to make the narrative more cohesive. For instance, in line 80 the authors introduce a matrix measure called the ‘logarithmic norm of the Jacobian’ which happens to be an important metric for the discussions that follow, but no concise mathematical form of it is provided to aid the readers.

o The authors have identified two quantitative tools to characterize contraction: contraction rate and the Jacobian of the network. However, in line 85 the authors have expressed one in terms of the other. Does that mean that the quantifications are interchangeable, and the analysis can be interpreted in terms of any one? Some explicit clarifications regarding this might be helpful.
o In line 86, the authors state that the idea of contraction analysis using Jacobian can be generalized to different metrics. What are some of these metrics? What are the metrics that can generate pure convergence of trajectories? What is the identity metric in line 138? A discussion along these lines including the mathematical expression of these metrics will enrich the study.

**Our Reply:**

**We agree that important quantifications need to be introduced in the text to make the narrative more cohesive and to improve overall clarity. We responded to the above three comments at the same time because we believe they stem from the same issue, as the reviewer points out: the lack of important mathematical definitions in the main text. We have edited the main text to hopefully resolve this issue.**

**In particular, we have added a mathematical definition of the logarithmic norm to the main text (beginning of Results section). Importantly, this also allows us to give the definition of ‘metric’ which hopefully resolves any confusion resulting from our omission of that definition. To reiterate briefly here, a metric is a symmetric, positive definite matrix which relates norms in one coordinate system to norms in another coordinate system. Every invertible coordinate transformation** $\boldsymbol{y=\theta x}$ **yields a metric** $\mathbf{M=}\boldsymbol{\theta}^{T}\boldsymbol{\theta}$**. This is because the (squared) norm of** $\mathbf{y}$ **is:**$\left| \left| \mathbf{y} \right| \right|^{2}\mathbf{=}\mathbf{y}^{\boldsymbol{T}}\mathbf{y}$ **=** $\mathbf{x}^{\boldsymbol{T}}\boldsymbol{\theta}^{T}\boldsymbol{\theta}\mathbf{x}\boldsymbol{=}\mathbf{x}^{\boldsymbol{T}}\mathbf{Mx}$. **One can see from this derivation that the identity metric simply corresponds to the original coordinate system (i.e** $\boldsymbol{\theta=I}$**).**

**We have also expanded the mathematical discussion to make clearer the relationship between the Jacobian, the contraction rate, and the logarithmic norm. To reiterate briefly here: if the logarithmic norm of the Jacobian is everywhere less than or equal to** $\boldsymbol{-c}$**, where** $\boldsymbol{c > 0}$**, this implies that the system is contracting with a contraction rate of** $\boldsymbol{c}$**. We have expanded and edited the manuscript to make this relationship clearer.**

o The types of perturbations considered for the study are not mentioned explicitly. Does the nature of convergence change depending upon the perturbation inflicted on the network?

**Our Reply:**

**We agree that this is an important question. The nature of convergence does not change depending upon the perturbation inflicted, besides the fact that if the larger the perturbation is (in norm) then the longer it may take for trajectories to reconvene once the perturbation is removed. Note that the *rate* of reconvening (i.e the contraction rate) does not change, however since the initial distance between trajectories at the time of removing the perturbation generally increases with the size of the perturbation, the time it takes for any two trajectories to get within** $\boldsymbol{\epsilon}$ **of each other also increases with the size of the perturbation. This fact relies on the assumption that the norm of the perturbation is not infinite, which is a realistic biological assumption. We have added a statement about this independence on nature of perturbation to the discussion in the main text.**

In line 211 the authors state that if alpha_i = 0, then W_max must be reduced to ensure equation 4 i.e., contraction in the network. My question here is in this case do you consider a densely connected network? Since, equation 4 states that if you are sparse enough you can have strong couplings irrespective of whether the synapses are static or dynamic.

**Our Reply:**

**The reviewer raised an important question. We do not make any assumptions on the sparsity on the network—the sparsity is captured by the** $\boldsymbol{p}_{\boldsymbol{i}}$**, which is the number of afferent synapses into neuron** $\boldsymbol{i}$**. Thus, for a densely connected network (for example, all-to-all neural connections), we would have** $\boldsymbol{p}_{\boldsymbol{i}}\boldsymbol{=N}$ **and W_max would have to be scaled appropriately to ensure contraction using this method. This is now clarified in the revised manuscript.**

o How should Figure 4 in the manuscript be interpreted? I understand that it points to the fact that E-I balance in networks (often employed by achieving E-I balance in subnetworks) facilitate stability and therefore convergence of trajectories under perturbation. But much of the preceding and succeeding discussion assumes prior knowledge of the matter. Introducing some key equations back into the text here will help justify the claims made.

**Our Reply:**

**We thank the reviewer for pointing this out—at some point during the writing process, the legend was cut-off. We apologize for this mistake. We have added the legend back. It illuminates why the concept of combining contracting systems is important for our analysis. To briefly summarize here, when analyzing the Hebbian plasticity described in equation (2) or the E-I balance later on, we conceptualize the overall system as a combination of two subsystems. Then we can use the known properties of combining contracting systems to derive our results.**

o Are there any neural mechanisms that have been studied experimentally to ensure stability and robustness in computation but cannot be explained solely through contraction analysis?

**Our Reply:**

**The reviewer raises an interesting question. It is difficult (indeed, perhaps impossible) to say what cannot be explained through contraction analysis alone without having a precise neural model in mind. But we do know that contraction is an extremely general framework for studying stability. In fact, it is known that the existence of a metric in which a system is contracting is a *necessary and sufficient* condition for exponential stability, which is the most ubiquitous type of stability. Experimentally, synaptic adaptation and homeostatic processes maintaining overall synaptic weights within the local network have been suggested as stabilizing factors for overall network activity. While we don’t analyze these specific cases in the interest of keeping the framework general, it seems likely our findings should apply to these cases in networks with more assumptions. We have added a sentence to the discussion highlighting our future plans to apply contraction analysis to these cases.**

• Minor Comments:
o Figure 1: Dimensionality of the cartoon state space. The text states that it is a (N+S) dimensional space for a network with N neural units and S dynamic synaptic weights, but the figure does not match up with it or perhaps need to be restructured. What is the index j here?
o Figure 2: Legends are not visible. Consider using larger font size.
o Line 157: There is no red trace in Figure 3A. Is it a typographical error or a missing trace?
o Caption for Figure 4 is incomplete.

**Our Reply:**

**We thank the reviewer for catching these typos. We have corrected all of them in the main text. For figure 1, the reviewer is correct that the synaptic index should run from 1 to j to S. For figure 2, we have expanded the legend. For line 157, this is a typographical error that has been corrected.**

o The Materials and Methods section does not add much in this version of the manuscript except where to find the relevant code. Consider expanding this section to include information that are vital to interpretation of the results.

**Our Reply:**

**This is a helpful suggestion. We believe that, after introducing key formulae from supplementary to the main text (as per the reviewer’s excellent earlier suggestion) we have covered the information which is vital to the interpretation of the results. We expanded the materials and methods sections to direct readers interested in the derivations of our findings to the appendix.**

o What is H in the supplementary section (Pg 13). Is it simply H = x_t x_t^T?

**Our Reply:**

**We thank the reviewer for their question. They are correct that this is the definition of H. It was deleted from the supplementary by mistake, and has been added back in.**
